# The Mass of Graviton and Its Relation to the Number of Information according to the Holographic Principle

**DOI:** 10.1155/2014/718251

**Published:** 2014-10-29

**Authors:** Ioannis Haranas, Ioannis Gkigkitzis

**Affiliations:** ^1^Department of Physics and Astronomy, York University, 4700 Keele Street, Toronto, ON, Canada M3J 1P3; ^2^Departments of Mathematics and Biomedical Physics, East Carolina University, 124 Austin Building, East Fifth Street, Greenville, NC 27858-4353, USA

## Abstract

We investigate the relation of the mass of the graviton to the number of information *N* in a flat universe. As a result we find that the mass of the graviton scales as $m_{\text{gr}}\propto 1/\sqrt{N}$. Furthermore, we find that the number of gravitons contained inside the observable horizon is directly proportional to the number of information *N*; that is, *N*_gr_ ∝ *N*. Similarly, the total mass of gravitons that exist in the universe is proportional to the number of information *N*; that is, $M_{\text{gr}}\propto \sqrt{N}$. In an effort to establish a relation between the graviton mass and the basic parameters of the universe, we find that the mass of the graviton is simply twice the Hubble mass *m*_*H*_ as it is defined by Gerstein et al. (2003), times the square root of the quantity *q* − 1/2, where *q* is the deceleration parameter of the universe. In relation to the geometry of the universe we find that the mass of the graviton varies according to the relation $m_{\text{gr}}\propto \sqrt{R_{\text{sc}}}$, and therefore *m*_gr_ obviously controls the geometry of the space time through a deviation of the geodesic spheres from the spheres of Euclidean metric.

## 1. Introduction

In Einstein's theory of general relativity, linearization of the field equations demonstrates that small perturbations of the metric obey a wave equation [1]. These small disturbances, referred to as gravitational waves, travel at the speed of light. However, some other gravity theories predict a dispersive propagation (see [2] for references). The most commonly considered form of dispersion supposes that the waves obey a Klein-Gordon-type equation that is given below:

(1) $$\begin{array}{c} \frac{1}{c^{2}}\left(\frac{\partial^{2}}{\partial t^{2}}-\nabla^{2}-{\left(\frac{m_{g}c^{2}}{\hbar }\right)}^{2}\right)\phi =0. \end{array}$$

Physically, the dispersive term is ascribed to the quantum of gravitation having a nonzero rest mass *m*_*g*_, or equivalently a noninfinite Compton wavelength *λ*_*g*_ = *h*/*m*_*g*_ *c*, where *ϕ* is the potential function of the gravitational field.

Moreover, several of today's theories like string theory, superstring theory, M-theory and loop quantum gravity, and quantum field theory predict the existence of graviton particles. In quantum field theory graviton is the elementary particle that mediates the gravitational force and is expected to be massless, and that is because the gravitational force itself has an infinite range. Furthermore, graviton must be a spin-2 boson, which results from the fact that the source of gravitation is the stress-energy tensor itself. Additionally, it can be shown that any massless spin-2 field could give rise to a force that is indistinguishable from gravitation because a massless spin-2 field must couple to the stress-energy tensor in the same way that the gravitational field does [3]. Therefore, this result suggests that if a massless spin-2 particle is discovered, it must be the graviton [1]. Thus graviton detection remains vital in the validation of the theories and also in the research that strives to unify quantum mechanics with general relativity. Although physicists normally speak as if bosons mediating the gravitational force exist, the extremely weak character of the gravitational interaction makes the detection of graviton an extremely hard issue. Recently, Dyson has suggested that “the detection of a single graviton may in fact be ruled out in the real universe” [4]. If researchers answer Dyson's question in an affirmative way, concluding that graviton detection is impossible, this will immediately raise issues in relation to the necessity of gravity quantization. However, attempts to extend the standard model with gravitons have run into serious theoretical difficulties at high energies (processes with energies close to or above the Planck scale) because of infinities arising due to quantum effects (in other words, gravitation is nonrenormalizable). Since classical general relativity and quantum mechanics are incompatible at such energies, from a theoretical point of view, the present situation is not tenable. Some proposed models of quantum gravity [5] attempt to address these issues, but these are speculative theories.

There are only a few conceivable sources of graviton production, like black hole decay, spontaneous emission of gravitons from neutral hydrogen, bremsstrahlung from electron-electron collisions in stellar interiors, and conservations of photons to gravitons by interstellar magnetic fields. Here we will only briefly touch upon the graviton production by black hole decay, which appears to be the most promising mechanism. Black holes of mass *M* possess a Hawking temperature that is equal to

(2) $$\begin{array}{c} T_{\text{BH}}=\frac{\hbar c^{3}}{8\pi k_{B}GM}, \end{array}$$

where *k*_*B*_ is the Boltzmann constant, *G* is the gravitational constant, *ħ* is Planck's constant, and *c* is speed of light. A primordial black hole with mass *M* ≤ 10^19^ kg can in principle evaporate gravitons of energy *E* ≥ 10 eV or higher and, with no observational constraints on the mass of the primordial black holes applied [6], can constitute most of the universe's dark matter. On the other hand, primordial black holes with mass *M* ≤ 10^12^ kg that emit particles with energy higher *E* ≥ 10^8^ eV would have already long been evaporated.

Recently, Finn and Sutton [7] have examined the binary pulsar, PSR B1913 + 16, of which the observed decay rate coincides with that expected from relativity to approximately 0.3% [7]. A nonzero graviton mass would upset this remarkable agreement altering the predicted orbital decay, implying an upper limit on the graviton mass. The authors have obtained a crude estimate on this bound using dimensional analysis. For a system with characteristic frequency *ω* one expects the effects of a graviton mass to appear at second order in *m*/*ω* [7]. For gravitational waves at twice the orbital frequency of PSR B1913 + 16, requiring (*m*/*ω*)^2^ < 0.003 implies an upper limit of order 10^−20^ eV/c^2^. This is comparable to the best limit from solar system observations, *m* < 0.44 × 10^−21^ eV/c^2^. Finn and Sutton [7] have examined an extension of linearized general relativity which includes a mass term for the graviton. They have chosen the unique mass term for which the wave equation of the linearized theory takes the standard form with an *h*-independent [7] source and for which the prepredictions of massless general relativity are recovered by setting *m* → 0 at the end of the calculations.

In recent papers by Novello and Neves [8] as well as Neves (2004) and Liao [9] the authors express a link between the cosmological constant and the graviton mass *m*_*g*_. Similarly, in Bousso [10] the author argues that the total observable entropy is bounded by the inverse of the cosmological constant. This holds for all space-times with a positive cosmological constant, including cosmologies dominated by ordinary matter and recollapsing universes [10]. Boussos' conjecture is largely influenced by Banks' idea of cosmological constant [11]. Moreover, in Mongan [12], the author examines a vacuum-dominated Friedmann universe asymptotic to a de Sitter space, with a cosmological event horizon that its area in Planck's units determines the maximum amount of information that will ever be available to any observer. Moreover, in a recent paper by Mureika and Mann [13], the authors examine the idea of information transfer between a test particle and the holographic screen in entropic gravity. The transfer respects both the uncertainty principle and causality, and a lower limit on the number of information bits in the universe relative to the mass may be derived. The corresponding limits indicate that particles travelling with the speed of light—photon and/or graviton—have a nonzero mass *m* ≥ 10^−68^ kg. Their result is found to be in excellent agreement with the current experimental mass bound of photon and graviton, suggesting that entropic gravity might be the result of a recent local symmetry that is softly broken [13]. In particular cosmological holography postulates that all the information content in our universe is encoded on its cosmological horizon. This proposal has been put forward by Smoot [14] and in short states that all possible past and future histories of our universe are encoded on its apparent horizon and by that making a connection. Moreover, the authors proceed by asking how much the universe as a whole, mass, and information included can tell us about its parts or the lightest possible mass of the elementary particles. The authors further claim that in entropic gravity there is a lower limit to the number of bits of holographic screen which provides the information transfer between the test particle and the screen, which obeys causality as well as the uncertainty principle.

Similarly, in Haranas and Gkigkitzis [15], the authors examine the Bekenstein bound of information number *N* and its relation to cosmological parameters in a universe, where in Gkigkitzis et al. [16] the authors use a recent result for the number of information *N* derived from Landauer's principle; they obtain an expression for the cosmological constant Λ. Finally, in Haranas and Gkigkitzis [17], the authors investigate the number of information *N* as related to the minimum quantum and gravitational masses in a vacuum-dominated universe. In this contribution we seek to investigate and understand the physics behind any possible relations that might result from the relation of the graviton mass to the surface area of the universe expressed in Planck units according to the holographic principle. As a result the number of information *N* on the horizon of the universe enters our calculation, and therefore the relation of the graviton mass to the number of information *N* will be established, via the cosmological constant lambda Λ dependence on the number of information. Moreover, we use Landauer's principle of information in relating the graviton mass to the mass of the universe and the mass of an elementary particle. Finally, we further investigate the relation of the graviton mass to the Ricci scalar and basic cosmological parameters of the universe.

## 2. The Mass of the Graviton

In a recent paper by Das (2014) the author uses the quantum Raychaudhuri equation to obtain the quantum corrected Friedmann equation for the range of graviton/photon. Similarly, with reference to Wesson [18], Novello [19], Tajmar [20], and Liao [9], we write that the proposed relation between the graviton mass and the cosmological constant is

(3) $$\begin{array}{c} \frac{1}{\lambda_{\text{gr}}^{2}}=\frac{2\Lambda }{3}=\frac{m_{\text{gr}}^{2}c^{2}}{\hbar^{2}}, \end{array}$$

where *ħ* is Planck's constant, *c* is the speed of light, and *λ*_gr_ is the graviton wavelength. This equation also follows from Einstein's linearized field equations which include the cosmological constant lambda Λ and also from the equations of motion for a massive spin-2 field that propagates in a de Sitter background. Using (1) we obtain that the mass of the graviton can be written as

(4) $$\begin{array}{c} m_{\text{gr}}=\frac{\hbar }{c}\sqrt{\frac{2\Lambda }{3}}. \end{array}$$

Equation (4) is actually the same equation as the one given in Wesson [18], in which $m_{\text{gr}}=m_{\text{wes}}/\sqrt{3}$. The dependence of the graviton mass on $\sqrt{\Lambda }$ is a property that remains valid not only for de Sitter but also for arbitrary background geometries as long as they stay in the setting of Einstein's second description of the equations of motion [19]. We can also construct a mass associated with lambda by using Planck's constant *ħ* and also the speed of light *c* (ibid., [19]). Following Novello [19] we write that the total mass of gravitons that exist in the universe is given by

(5) $$\begin{array}{c} M_{\text{gr}}=m_{\text{gr}}N_{\text{gr}}=\frac{c^{3}}{G\hbar \Lambda }\frac{\hbar }{c}\sqrt{\frac{2\Lambda }{3}}=\frac{c^{2}}{G}\sqrt{\frac{2}{3\Lambda }}, \end{array}$$

where *N*_gr_ is the total number of gravitons contained inside the observable horizon and it is equal [19] to

(6) $$\begin{array}{c} N_{\text{gr}}=\frac{c^{3}}{G\hbar \Lambda }=\frac{1}{\Lambda }=\frac{\Lambda_{\max}}{\Lambda }, \end{array}$$

where *ℓ*_*P*_ is the Planck length and Λ_max⁡_ = *c* ^3^/*Għ* is the maximum value of the cosmological constant as it is defined in [15]. Therefore we write the total number of gravitons in the universe in the following way:

(7) $$\begin{array}{c} N_{\text{gr}}=\frac{\Lambda_{\max}}{c}\left(\sqrt{\frac{2}{3\Lambda }}\right)\hbar . \end{array}$$

## 3. The Relation of the Graviton Mass to the Number of Information

The holographic principle indicates a possible nonlocality mechanism in any vacuum-dominated Friedmann universe. To be more precise, a holographic nonlocal quantum mechanical description can be possible for a finite amount of information in a closed vacuum-dominated universe. Today's theories assume that the universe began by a quantum fluctuation from nothing, underwent inflation, and became so large that it is locally almost flat and that since the inflationary era the vacuum energy density of the universe is constant. This is the case of the existence of a nonzero cosmological constant Λ. More information of such a universe arising in quantum cosmological way is presented in Mongan [21]. Systems that dynamically evolve in time not only transform but also process information. Using the relation given in Haranas and Gkigkitzis [15, 17] we can write the cosmological constant as a function of the number of information to be

(8) $$\begin{array}{c} \Lambda =\frac{3\pi }{Nl_{P}^{2}\ln2}=\frac{3\pi }{N\ln2}\Lambda_{\max}. \end{array}$$

Substituting (8) into (2) we obtain that

(9) $$\begin{array}{c} m_{\text{gr}}=\frac{\hbar }{c}\sqrt{\frac{2\pi }{Nl_{P}^{2}\ln2}}=\frac{\hbar }{cl_{P}}\sqrt{\frac{2\pi }{N\ln2}}=\xi_{0}\frac{m_{\text{Pl}}}{\sqrt{N}}, \end{array}$$

where *ξ*_0_ = (2*π*/ln⁡2)^1/2^ ≈ 3.010 and *m*_Pl_ = (*ħc*/*G*)^1/2^ is the Planck mass; therefore we see that $m_{\text{gr}}\propto 1/\sqrt{N}$. Similarly, the total number of gravitons contained inside the observable horizon can be written as a function of the number of information *N* in the following way:

(10) $$\begin{array}{c} N_{\text{gr}}=\frac{c^{3}}{G\hbar }\frac{Nl_{P}^{2}\ln2}{3\pi }=\frac{\ln2}{3\pi }N, \end{array}$$

or in other words *N*_gr_ ∝ *N*. Therefore, the total graviton mass in the universe is equal to

(11) $$\begin{array}{c} M_{\text{gr}}=m_{\text{gr}}N_{\text{gr}}=\eta_{0}m_{\text{Pl}}\sqrt{N}, \end{array}$$

where *η*_0_ = (1/3)(2ln⁡2/*π*)^1/2^ and therefore $M_{\text{gr}}\propto \sqrt{N}$. Similarly, using (9), we obtain that the number of information *N* is given by

(12) $$\begin{array}{c} N=\frac{2\pi }{\ln2}{\left(\frac{m_{\text{Pl}}}{m_{\text{gr}}}\right)}^{2}. \end{array}$$

Moreover, from (11), we obtain a second expression for the number of information *N* that reads

(13) $$\begin{array}{c} N=\frac{9\pi }{2\ln2}{\left(\frac{M_{\text{gr}}}{m_{\text{Pl}}}\right)}^{2}. \end{array}$$

As a result we find that the number of information *N* can be expressed as the square of the ratio of two fundamental masses, namely, the Planck mass and the mass of the graviton or the square of the total number of gravitons in the universe over the Planck mass.

## 4. Landauer's Principle and the Mass of the Graviton

In relation to the laws of physics we say that they determine the amount of information that a given system can register (i.e., number of bits or nats) as well as the number of elementary logic operations that the given system can perform (i.e., number of operations). With reference to Landauer [16, 22, 23] we can say that information is physical, and that also all information is registered and processed by physical systems. Physical systems can be described in terms of information, and information processing is related to the system description by physical laws. Landauer's principle is a physical principle pertaining to the lower theoretical limit of energy consumption of a computation. Landauer postulated that any “logically irreversible manipulation of information, such as the erasure of a bit or the merging of two computation paths, must be accompanied by a corresponding entropy increase in non-information-bearing degrees of freedom of the information processing apparatus or its environment” [24]. Landauer's principle asserts that there is a minimum possible amount of energy required to change one bit of information, known as the Landauer limit, and it is equal to [25]

(14) $$\begin{array}{c} E_{\min}=k_{B}T\ln2, \end{array}$$

where *k*_*B*_ = 1.38 × 10^−23^ J/K is Boltzmann's constant and *T* is the temperature of the circuit. Therefore Landauer's energy formula can be written in terms of the information number *N* in the following way:

(15) $$\begin{array}{c} E=Nk_{B}T\ln2. \end{array}$$

Recent experimental studies provide further evidence that Landauer's prediction is true. For example, the equivalence between information and energy can be interpreted using the results obtained in recent experiment by Funo et al. [26].

In their experiment the authors have shown that entanglement can produce an increase or gain of thermodynamic work, where the gain is determined by the change of the information content. Similarly, Bérut et al. [27] have shown that there is a link between information theory and thermodynamics. Landauer's principle is a simple consequence and its logic emanates directly from the second law of thermodynamics. The law states that the entropy of a closed system cannot decrease at the same time with the corresponding temperature. Therefore, if one nat of information is lost during a computation, the amount of entropy generated is at least *k*_*B*_ln⁡2, and therefore the energy emitted in the environment is *E* ≥ *k*_*B*_ *T*ln⁡2. Landauer's principle has been accepted as a physical law, but it has also been challenged by Shenker [28] and Norton [29] and defended by Bennett [24] and Ladyman et al. [30].

Next, with reference to Alfonso-Faus and Fullana i Alfonso [31] and Gkigkitzis et al. [16], the authors say that “all physical systems of mass *M* and energy *Mc* ^2^ are equivalent to an amount of information in number of bits” of the order of

(16) $$\begin{array}{c} N≅\left(\frac{Mc^{2}}{\hbar H}\right), \end{array}$$

where *M* is the mass of the system, *c* is the speed of light, *ħ*  is Planck's constant, and *H* is the Hubble constant.

In Alfonso-Faus and Fullana i Alfonso [31] the authors claim that (3) is of “universal validity.” They further say that the unit of energy that should be taken as the minimum quantum of energy is *ħH*. This implies that the relativistic energy of a mass *M* has *N* times this minimum quantum of energy *NħH*, where *N* is the number of information in nats. In other words the product *NħH* corresponds to the energy of all the information number *N* carried by the system. Thus, the expression for the cosmological constant lambda Λ obtained in Gkigkitzis et al. [16] is used below:

(17) $$\begin{array}{c} \Lambda =\frac{3\pi }{\ln2}\left(\frac{Hc}{M_{u}G}\right), \end{array}$$

where *M*_*u*_ is the total mass of the universe and *H* is the Hubble parameter, and substituting (16) in (2) and simplifying we obtain that

(18) $$\begin{array}{c} m_{\text{gr}}=\xi_{0}\sqrt{\frac{\hbar^{2}H}{GcM_{u}}}=\xi_{0}\left(\frac{m_{\pi }^{3/2}}{M_{u}}\right)=\xi_{0}{\left(\frac{m_{\pi }^{3}}{M_{u}}\right)}^{1/2}, \end{array}$$

where *m*_*π*_ = (*ħ* ^2^ *H*/*cG*)^1/3^ is the mass of the pion as it is given by Weinberg [32], and therefore we can write the mass of the universe *M*_*u*_ in the following way:

(19) $$\begin{array}{c} M_{u}=\xi_{0}^{2}\left(\frac{m_{\pi }^{3}}{m_{\text{gr}}^{2}}\right), \end{array}$$

or in other words as the ratio of two fundamental particle masses in the universe.

## 5. Time Dependence of the Graviton Mass, Ricci Scalar, and Information Number

In order to investigate a possible relation of the graviton mass to time *t* let us now consider a flat universe, that is, *k* = 0, where *k* is the curvature constant and density parameter *Ω* = 1. Following Gkigkitzis et al. [16] we can write that in a flat universe the number of information *N* evolves as a function of time in the following way:

(20) $$\begin{array}{c} N\left(t\right)=\frac{4\pi }{\ln2}{\left(\frac{c}{l_{P}}\right)}^{2}t^{2}=\left(\frac{4\pi }{\ln2}\right){\left(\frac{t}{t_{P}}\right)}^{2}. \end{array}$$

The bound is not fixed but rather grows as time progresses and the horizon expands and encompasses more particles [33]. The expression above is in agreement with the work of Lloyd [23] and Davies [33] where the authors predict that in a flat universe *N* ∝ *t* ^2^. Substituting (20) in (9) we obtain that

(21) $$\begin{array}{c} m_{\text{gr}}={\left(\frac{\ln2}{8\pi }\right)}^{1/2}m_{\text{Pl}}\left(\frac{t_{\text{Pl}}}{t}\right), \end{array}$$

and therefore we find that the mass of the graviton in a flat universe evolves as *m*_gr_ ∝ 1/*t*. Using (21) we find that in a flat universe at the moment where *t* = *t*_0_ where *t*_0_ = 4.346 × 10^17^ s [24] is the age of the universe the mass of the graviton becomes

(22) $$\begin{array}{c} m_{\text{gr}}={\left(\frac{\ln2}{8\pi }\right)}^{1/2}m_{\text{Pl}}=8.777\times 10^{-62}m_{\text{Pl}}\left[\text{kg}\right]. \end{array}$$

In order to investigate the relation of the graviton mass with fundamental cosmological parameters let us look at Haranas and Gkigkitzis [34]. In this paper the authors derive the Ricci scalar that is defined by [35, 36]

(23) $$\begin{array}{c} R_{\text{sc}}=-\left(\frac{6\ddot{R}}{c^{2}R}+\frac{6{\dot{R}}^{2}}{c^{2}R^{2}}+\frac{6k}{R^{2}}\right). \end{array}$$

*R* is the scale factor of the universe. In a flat universe the resulting Ricci scalar can be written as a function of the number of information number *N* in the following way to be [34]

(24) $$\begin{array}{c} R_{\text{sc}}=\frac{6\pi }{\ln2}\frac{\left(q-\Omega \right)}{l_{P}^{2}N}, \end{array}$$

where *q* and *Ω* = 1 are the deceleration and density parameters of the flat universe. Solving (24) for the deceleration parameter of the universe we obtain

(25) $$\begin{array}{c} q=\Omega +\frac{\ln2}{6\pi }l_{P}^{2}R_{\text{sc}}N. \end{array}$$

Next, in a flat universe filled with nonrelativistic matter, that is, *p* = 0, corrected for the graviton contribution, we have that the deceleration parameter given by [37]

(26) $$\begin{array}{c} q=-\frac{\ddot{R}}{RH^{2}}=\frac{1}{2}+\frac{1}{4H^{2}}{\left(\frac{m_{\text{gr}}c^{2}}{\hbar }\right)}^{2}. \end{array}$$

Therefore, equating (25) and (26), we obtain that the mass of the graviton is given by

(27) $$\begin{array}{c} m_{\text{gr}}^{2}=\frac{4\hbar^{2}H^{2}}{c^{4}}\left[\Omega -\frac{1}{2}+\frac{\ln2}{6\pi }l_{P}^{2}R_{\text{sc}}N\right], \end{array}$$

where the quantity *ħH*/*c* ^2^ = *m*_Hub_ is the Hubble mass defined in [38]. In the present era we have that *m*_Hub_^0^ = *ħH*_0_/*c* ^2^ = *h*(3.8 × 10^−66^) g≅2.7 × 10^−66^ g when *h* = 0.71 [38]. Equation (27) gives the mass of the graviton in terms of the Humble parameters *H*, the density parameter *Ω*, the Planck length *ℓ*_*P*_^2^, the Ricci scalar *R*_sc_, and finally the number of information number *N*. Using (5) in Haranas and Gkigkitzis [34] and eliminating the number of information *N*, that is, *N* = 6*π*(*q* − *Ω*)/ln⁡2*ℓ*_*P*_^2^ *R*_sc_, we obtain the following expression for the mass of the graviton to be

(28) $$\begin{array}{c} m_{\text{gr}}=\frac{2\hbar H}{c^{2}}{\left(q-\frac{1}{2}\right)}^{1/2}=2m_{\text{Hub}}{\left(q-\frac{1}{2}\right)}^{1/2}. \end{array}$$

Similarly, we find that the mass of the graviton is related to the Ricci scalar *R*_sc_ in the following way:

(29) $$\begin{array}{c} m_{\text{gr}}=\frac{2\hbar }{c}{\left[\frac{\left(q-1/2\right)}{3\left(q-\Omega \right)}R_{\text{sc}}\right]}^{1/2}, \end{array}$$

where *R*_*H*_ is the universe horizon, as well as to deceleration parameter *q*. Using the fact that the graviton range is given by *λ*_gr_ = *ħ*/*m*_gr_ *c* [38] we obtain an expression for the Ricci scalar as a function of graviton range and the cosmological parameter *q* to be

(30) $$\begin{array}{c} R_{\text{sc}}=\frac{3}{4\lambda_{\text{gr}}^{2}}\frac{\left(q-\Omega \right)}{\left(q-1/2\right)}. \end{array}$$

Next, following Gershtein et al. [37] we write the expression for the time that the universe expands from a maximal density to a minimal density dominance and is determined by the stage of the nonrelativistic matter dominance to be

(31) $$\begin{array}{c} t_{\max}≅\sqrt{\frac{2}{3}}\frac{\pi \hbar }{m_{\text{gr}}c^{2}}. \end{array}$$

Therefore, (31) can also be related to the number of information *N* via the relation of the graviton mass to the number of information, and therefore we find that

(32) $$\begin{array}{c} t_{\max}≅\sqrt{\frac{\pi \ln2}{3}}t_{\text{Pl}}\sqrt{N}. \end{array}$$

Solving (32) for the number of information *N* we obtain that

(33) $$\begin{array}{c} N=\frac{3}{\pi \ln2}{\left(\frac{t}{t_{\text{Pl}}}\right)}^{2}. \end{array}$$

Our result is in agreement with that of Lloyd [23] and Davies [33] where the author predicts that this is equal to the maximum number of bits registered by the universe using matter, energy, and gravity, and it is found with the help of the Bekenstein bound and the holographic principle to the universe as a whole. It is given by the square of the ratio of the age of the universe to that of Planck time. Similarly, using (9) for the total mass of the gravitons, we find that the number of information *N* can also be written in the following way:

(34) $$\begin{array}{c} N=\frac{9\pi }{2\ln2}{\left(\frac{M_{\text{gr}}}{m_{\text{Pl}}}\right)}^{2}. \end{array}$$

## 6. Discussion and Numerical Results

In this paper we have considered an expression for the mass of the graviton as it is given by Novello [19]. Novello's expression depends on the cosmological constant lambda Λ. Using the result in Haranas and Gkigkitzis [15, 17] we find a relation of graviton mass *m*_gr_  to the number of information *N*. As a result we find that the mass of the graviton is inversely proportional to the number of information *N*; that is, $m_{\text{gr}}\propto 1/\sqrt{N}$. Furthermore we find that the number of gravitons contained inside the observable horizon is directly proportional to the number of information *N*; that is, *N*_gr_ ∝ *N*. Similarly, the total mass of gravitons that exist in the universe is proportional to the number of information *N*; that is, $M_{\text{gr}}\propto \sqrt{N}$. We find two different expressions for the number of information *N*, one that is given as the square of the ratio of the Planck mass to the mass of the graviton, that is, *N* ∝ (*m*_*P*_/*m*_gr_)^2^, and another that is equal to the square of the ratio of the total graviton mass in the universe over the Planck mass, that is, *N* ∝ (*M*_gr_/*m*_*P*_)^2^. Next, using a recent definition for the number of information *N* resulting from Landauer's principle, we find that the mass of the graviton can be expressed as the square root of third power of the pion mass *m*_*π*_^3^ over the mass of the universe *M*_*u*_, which implies that the mass of the universe is further equal to the third power of the pion mass *m*_*π*_^3^ over the square of the Planck mass *m*_Pl_^2^. Moreover we find that in a flat universe the graviton mass varies according to *m*_gr_ ∝ 1/*t*, and furthermore we find that at time *t* = *t*_0_ (where *t*_0_ is age of the universe) the mass of the graviton is *m*_gr_ = 8.777 × 10^−62^ *m*_Pl_ = 1.909 × 10^−69^ kg. This order of magnitude is in agreement with the result given in Gershtein et al. [38] where the authors predict that *m*_gr_ = 3.2 × 10^−66^ g to 95% confidence level. Similarly DGP (Dvali-Gabadadze-Porrati) constraints predict that the mass of the graviton falls in the range *m*_gr_≅10^−67^ − 10^−69^ kg [13].

In an effort to establish a relation of the mass of graviton to basic parameters of the universe, wefind that the mass of the graviton is simply twice the Hubble mass *m*_*H*_ as it is defined by Gershtein et al. [38], times the square root of the quantity *q* − 1/2, where *q* is the deceleration parameter of the universe. In reference to the geometry of the universe we find that the mass of the graviton varies according to the relation $m_{\text{gr}}\propto \sqrt{R_{\text{sc}}}$ or *m*_gr_^2^ ∝ *R*_sc_ and therefore *m*_gr_ obviously controls the geometry of the space time through a deviation of the geodesic spheres from the spheres of Euclidean metric. In general relativity, the scalar curvature is the Lagrangian density for the Einstein-Hilbert action. Therefore, the graviton mass *m*_gr_ determines the curvature *R*_sc_ since *R*_sc_ ∝ *m*_gr_^2^ and therefore the Einstein-Hilbert action itself depends on the graviton mass. Similarly, we obtain that the Ricci scalar scales as *R*_sc_ ∝ 1/*λ*_gr_^2^, and in a similar way the action itself can also depend upon the range of the graviton *λ*_gr_. Furthermore, the time interval taken for the universe expansion from a maximum to a minimum density varies as the square root of the number of information, that is, $t_{\max}\propto \sqrt{N}$, resulting in an expression for the number of information as a function of time *t* that agrees with that derived by Lloyd [23]; namely, *N* = (3/*π*ln⁡2)*N*_Lloyd_. Using (2) and (6) we find that number of information *N* associated with the mass of the graviton is given by

(35) $$\begin{array}{c} N=\left(\frac{3\pi \hbar^{2}}{\ln2c^{2}l_{\text{Pl}}^{2}m_{\text{gr}}^{2}}\right)=\frac{2\pi }{\ln2}{\left(\frac{m_{\text{Pl}}}{m_{\text{gr}}}\right)}^{2}\approx 4.20\times 10^{122}, \end{array}$$

where the mass of the graviton is taken to be *m*_gr_ = 3.2 × 10^−66^g [38]. This is a rather curious result which suggests that the number of information bits *N* associated with the graviton mass is of the same order of magnitude as the numbers appearing in cosmology. For example, Funkhouser [39] finds a new large number of coincidences as well as a scaling law for the cosmological constant and other quantities which are also investigated. As a result the author claims that the pure numbers originate naturally from basic ratios of fundamental parameters and they do not require arbitrary powers of coefficients [39]. In a similar paper [17] we have shown that the number of information *N* is associated with the cosmological constant lambda Λ and is equal to 4.661 × 10^122^, a number in agreement with that given in Funkhouser [39]. This is still two orders of magnitude smaller than the number of information that can fit in the universe to which a physical meaning can be attributed. This is a huge number but still small to compare various numbers, one of which is called Graham number [40]. Finally the entropy of the graviton mass can be estimated using the formula

(36) Sgr=kBlog2(2N)=(2πln⁡2)(mPlmgr)2kB≈4.191×10122kB≈6.0×1099 J/K.

The calculated entropy is of the same order of magnitude as the entropy within the cosmic horizon calculated when matter within is taken into account and has been recently given in a paper by Egan and Lineweaver [41] to be equal to (2.88 ± 0.16) × 10^122^ *k*_*B*_≅4.0 × 10^99^ J/K. Following Novello [19] we have that *N*_gr_ = *c* ^3^/*Għ*Λ = 1/*ℓ*_*P*_^2^Λ = Λ_max⁡_/Λ, where *N*_gr_ is the total number of gravitons in the universe, and therefore the total entropy due to the total number of gravitons is given by

(37) $$\begin{array}{c} S_{\text{gr}}\left(\text{tot}\right)=\left(\frac{2\pi }{\ln2}\right)\frac{k_{B}c^{4}}{\Lambda G^{2}m_{\text{gr}}^{2}}. \end{array}$$

Using (6) and (7) along with the fact that *ℓ*_*P*_^2^ = *Għ*/*c* ^3^ in (32) we find that the entropy due to the total number of gravitons in the universe as a function of the number of information *N* is given by the following simplified expression:

(38) $$\begin{array}{c} S_{\text{gr}}\left(\text{tot}\right)=\left(\frac{\ln2}{3\pi }\right)k_{B}N^{2}=1.015\times 10^{-24}N^{2}. \end{array}$$

Moreover in a flat universe using (18) we obtain that the entropy due to the total number of gravitons in the universe evolves in time in the following way:

(39) $$\begin{array}{c} S_{\text{gr}}\left(\text{tot}\right)=\left(\frac{16\pi }{3\ln2}\right)k_{B}{\left(\frac{t}{t_{\text{Pl}}}\right)}^{4}. \end{array}$$

During a very early era and in particular when *t* = *t*_Pl_ the total entropy due to the total number of gravitons takes the values

(40) $$\begin{array}{c} S_{\text{gr}}\left(\text{tot}\right)=\left(\frac{16\pi }{3\ln2}\right)k_{B}=3.335\times 10^{-22}\;\text{J}/\text{K}, \end{array}$$

where *k*_*B*_ = 1.38 × 10^−23^ J/K and $t_{p}=\sqrt{\hbar G/c^{5}}=5.391\times 10^{-44}$ s. Similarly, in the present era when *t* is the age of the universe, *t* = *t*_age_ = 13.798 by =4.387 × 1017 s [24], we obtain that the total entropy due to the total number of gravitons is given by

(41) $$\begin{array}{c} S_{\text{gr}}\left(\text{tot}\right)=3.336\times 10^{222}\;\text{J}/\text{K}. \end{array}$$

Finally, using the relation given in Mureika and Mann [13], we write that the graviton mass satisfies the following relation:

(42) $$\begin{array}{c} m_{\text{gr}}\geq 16\pi^{2}\left(\frac{M_{u}}{N}\right), \end{array}$$

where *M*_*u*_ is the mass of the universe and *N* = *N*_*u*_ is the number of events or operations that could have occurred in the age of the universe *t* = *t*_age_. Using that, the maximum number of bits using gravitational degrees of freedom as well as conventional matter and energy is equal to the maximum number of elementary operations [23] and using an expression given in Gkigkitzis et al. [16] we write that

(43) $$\begin{array}{c} N\left(t\right)≅N_{\text{ops}}=\frac{4\pi }{\ln2}{\left(\frac{t}{t_{\text{Pl}}}\right)}^{2}, \end{array}$$

and after substituting in (42) we obtain that

(44) $$\begin{array}{c} m_{\text{gr}}\geq 4\pi \ln2\left(t_{\text{Pl}}^{2}H^{2}\right)M_{u}. \end{array}$$

Next substituting for the Planck time *t*_Pl_ = (*Għ*/*c* ^5^)^1/2^ and for the mass of the universe as it is given in Haranas and Gkigkitzis [15] and also Valev [42], namely, *M*_*u*_ = *c* ^3^/*GH*, and simplifying we obtain that

(45) $$\begin{array}{c} m_{\text{gr}}\geq 4\pi \ln2\left(\frac{\hbar H}{c^{2}}\right)\geq 4\pi \ln2m_{\text{Hub}}, \end{array}$$

where *m*_Hub_ = *ħH*/*c* ^2^ is defined in Gershtein et al. [38], to be the Hubble mass. Following Sivaram [43] we define the gravitational self-energy of the graviton to be *E*_GSE_ = *ħH* (45) which can be further written as

(46) $$\begin{array}{c} m_{\text{gr}}\geq 4\pi \ln2\left(\frac{E_{\text{GSE}}}{c^{2}}\right)\leq 4\pi \ln2m_{\text{GSE}}, \end{array}$$

where *m*_GSE_ is the corresponding gravitational self-mass of the graviton. In Valev [44] the author gives a theoretical estimation of the graviton mass based on the assumption that the Compton wavelength of the graviton is close to the Hubble distance *c*/*H*, which produces a value of the graviton mass that is given by the relation *m*_gr_ ≈ *ħH*/*c* ^2^.

## 7. Conclusions 

In this paper we investigate the relation of the graviton mass to the number of information *N* in the universe, and an *N* ^−1/2^ dependence has been found. Similarly, we find that the total number of gravitons inside the horizon of the universe is proportional to the number of information *N*. Furthermore, using Landauer's principle, we obtain that the mass of the graviton can also be expressed as the one-third power of the ratio of the mass of the pion over the mass of the universe, from which we obtain that the mass of the universe is related to the cube of the mass of the pion over the square of the graviton mass. Moreover, we find that the evolution of the graviton has an inverse time dependence; that is, *m*_gr_ ∝ 1/*t*. Finally, in relation to the geometry of the universe, we find that the mass of the graviton is related to the Ricci scalar in the following way: $m_{\text{gr}}\propto \sqrt{R_{\text{sc}}}$, where at the same time the Ricci scalar depends on the inverse of the graviton range according to the relation *R*_sc_ ∝ 1/*λ*_gr_^2^.
